# Feasibility of automated registration of Patient-Reported Outcome Measures (PROMs) for inguinal hernia surgery : a single-centre pilot study

**DOI:** 10.1007/s10029-026-03822-4

**Published:** 2026-10-15

**Authors:** R. R. Meuzelaar, H. V. Smit, F. P.J. den Hartog, A. H.W. Schiphorst, P. J. Tanis, J. P.J. Burgmans

**Affiliations:** 1 Department of Surgery, Diakonessenhuis, Utrecht, The Netherlands; 2 Department of Surgery, Erasmus Medical Centre, Rotterdam, The Netherlands

**Keywords:** Inguinal hernia surgery, PROMs, Automated registration, Response rate, Quality of care

## Abstract

**Purpose:**

National registries have the potential to improve quality of inguinal hernia surgery by monitoring provided care and comparing outcomes. Patient-reported outcome measures (PROMs) are essential for assessing quality as they capture key outcomes directly from the patient's perspective. This pilot study evaluated feasibility of automated PROMs registration up to 2 years postoperatively with minimal administrative burden in a high-volume hernia clinic.

**Methods:**

Adults who underwent elective inguinal hernia repair and received PROMs questionnaires between January 18, 2023, and August 25, 2025, were prospectively included. Patients received secure email invitations to complete questionnaires through the hospital’s patient portal at baseline and at 6–8 weeks (short-term), 3 months, 1 year, and 2 years postoperatively. PROMs included pain, functional recovery, satisfaction, and hernia recurrence. The primary outcome was the response rate at different follow-up time points (target rate: 60%). Key secondary outcomes included physician-assisted response rates and responder versus non-responder differences.

**Results:**

A total of 3,628 patients were included. Preoperative and short-term response rates were 77.7% and 63.2%, respectively. Postoperative response rates declined to 47.7% (3 months), 44.2% (1 year), and 43.8% (2 years). Younger age and a higher burden of comorbidities were associated with non-response.

**Conclusion:**

This study demonstrated feasibility of automated PROMs at baseline and short-term follow-up, but highlights the challenge of declining response rates over time and representativeness of the responding population. Integrating this approach into a national registry could improve data collection and overall efficiency. Strategies to optimize response rates and representativeness should be explored.

**Supplementary Information:**

The online version contains supplementary material available at https://doi.org/10.1007/s10029-026-03822-4.

## Introduction

Over the past 40 years, substantial advancements have been achieved in inguinal hernia surgery, including the introduction of mesh repairs and laparoendoscopic techniques, leading to improved surgical outcomes thereby enhancing overall quality of care [1–3]. These advancements were primarily driven by randomized controlled trials (RCTs), which offer high internal validity [4]. However, external validity might be limited due to strict eligibility criteria, patient refusal, rather standardized interventions, selection of dedicated participating centres, and lack of head-to-head comparisons of every available intervention. In addition, these trials cause significant logistical burden on investigators, are frequently not completed, take years to deliver results, and are associated with high costs.

Moreover, inguinal hernia repair is a high-volume procedure, accounting for 30,000 annual repairs in the Netherlands alone [5]. Given this scale, even small improvements in surgical practices can have a substantial impact on overall patient care. However, as the focus has shifted from major breakthroughs towards continuous quality improvement, detecting these subtle refinements require large population-based datasets. In light of the limitations related to RCTs, registry-based studies have become essential for assessing and improving healthcare quality as an adjunct to clinical trials. By systematically collecting real-world data, registries have the potential to reflect routine daily practice across all patient groups, thereby strengthening evidence-based medicine and improving patient outcomes [6].

Currently, the Dutch Inguinal Hernia Audit (DIHA) is being established to monitor and improve national quality standards by benchmarking surgical techniques and mesh types, and analysing interhospital variability [7]. Clinical data are collected via an automated system, reducing workload and administrative burden. These data can potentially contribute to enhanced surgical quality, however, it lacks the patient’s perspective on the most important complications of inguinal hernia repair: chronic postoperative inguinal pain (CPIP) and hernia recurrence [8]. Integrating patient-reported outcome measures (PROMs) could offer a more patient-centred perspective on the effectiveness of surgery, including quality of life and physical functioning, providing valuable insights that clinical data alone cannot capture [9].

The use of lengthy standardized questionnaires, combined with manual data entry of paper-based responses, makes PROMs collection time consuming for both patients and healthcare staff. Automated collection offers a potential solution: Patients undergoing inguinal hernia repair receive secure, automated emails to complete short PROMs questionnaires at fixed time intervals. These responses are subsequently synchronized with the electronic medical record, allowing healthcare professionals to assess the responses during routine clinical practice. Additionally, the aggregated output can be used for auditing and research. The main advantage of this automated approach is that it significantly minimizes the administrative burden.

To evaluate the feasibility of this solution, the automated PROMs collection system was integrated into a high-volume hernia clinic, with the potential for national implementation within the DIHA. Before widespread adoption, it is crucial to assess patients’ willingness to complete these questionnaires. This pilot study aimed to evaluate the feasibility of incorporating automated PROMs up to 2 years postoperatively in routine inguinal hernia care without the involvement of healthcare personnel or additional administrative burden. The primary objective was to determine the response rate at each time point during follow-up.

## Methods

### Study design

This study retrospectively analysed data from a prospective PROMs database, including all inguinal hernia patients treated in a high-volume hernia clinic between January 18, 2023 (marking the implementation of the updated version of the PROMs questionnaire), and August 25, 2025 (the date of database extraction). All adults undergoing a totally extraperitoneal (TEP) repair or an open procedure with mesh (Lichtenstein) were included. Only elective hernia repairs were considered eligible, as patients treated in an emergency setting could not complete the baseline questionnaire before surgery. Similarly, patients were excluded if they missed either the preoperative or postoperative PROMs invitation, because these were sent separately and this prevented them from the opportunity to complete the questionnaire. Other techniques than TEP or Lichtenstein were also excluded. Ethical approval was obtained from the Institutional Review Board. The study was conducted in accordance with the Strengthening the Reporting of Observational Studies in Epidemiology (STROBE) guidelines [10].

## Routine surgical care

The preferred technique was TEP repair, unless contraindications were present (recurrent hernia after TEP repair, previous preperitoneal surgery or general anaesthesia was not possible). Each surgeon surpassed the TEP learning curve and had performed over 500 procedures. TEP repairs were performed under general anaesthesia using a 10 × 15 cm (synthetic, nonabsorbable, heavyweight, microporous) polypropylene mesh [11], whereas Lichtenstein repairs were conducted under spinal anaesthesia with a 6 × 11 cm (synthetic, partially absorbable, lightweight, macroporous) polypropylene mesh [12]. Patients were typically discharged on the same day, unless adverse events prevented early discharge. During the first postoperative week, patients were advised to refrain from strenuous activities and to take analgesics (paracetamol) in case of any level of pain. Follow-up was conducted via a telephone consultation at 6 to 8 weeks after surgery [13].

### PROMs

The PROMs questionnaires were developed based on consensus among a team of Dutch inguinal hernia experts. An initial draft was evaluated and refined using the results of a national survey that assessed support, obstacles, and potential areas for improvement within the DIHA [7]. The current version consists of questions addressing topics such as pain, return to work and exercise, hernia recurrence, and patient satisfaction about their treatment. To improve completion rates and minimize participant burden, PROMs questionnaires were designed to be concise and specifically focused on relevant topics. This approach aimed to reduce the likelihood of patients disengaging due to lengthy questionnaires. To further shorten the survey, dynamic questions were included, allowing for a tailored experience based on individual responses. For example, if a patient reported no complaints, the questionnaire was automatically shortened, ensuring it remained as brief as possible while still collecting the necessary data. Fixed questions were included for consistency across all participants. For this article, the PROMs questionnaires were translated into English, with details provided in Online Resource 1 (eTable 1).

After development, the PROMs were integrated into routine patient care. Patients who objected to the use of these data for research purposes could opt out by submitting an objection, which resulted in the exclusion of their data from future studies.

Patients received a secure email invitation to complete the PROMs questionnaires through the hospital’s patient portal at baseline (preoperative) and at 6–8 weeks (short-term), 3 months, 1 year, and 2 years postoperatively (Table 1). Access was only possible via the Digital Identification (DigiD), the national online authentication system established by the government [14]. DigiD enables residents to securely access government services, healthcare portals, and other official organizations online. The answers were automatically stored in the hospital’s electronic medical record. Preoperative PROMs were linked to the initial outpatient clinic appointment and were automatically dispatched once the appointment was scheduled. Postoperative PROMs were linked to the surgical procedure and the uploading of the operative report served as the trigger for their automated distribution.

**Table 1:** Schematic overview of PROMs questionnaire domains at each follow-up moment

	Baseline^a^	6–8 weeks	3 months	1 year	2 years
Pain	✓	✓	✓	✓	✓
Physical functioning	✓	✓			
Recurrence		✓	✓	✓	✓
Satisfaction			✓		
Surgical history LAQ^b^	✓				

^a^ Reminders were sent if the questionnaire was not completed

^b^ *LAQ* lower abdominal quadrant

A different response window was applied for each questionnaire. The baseline questions had to be answered any time before surgery. For the PROMs at 6–8 weeks, patients had 7 days to submit their responses. Three months postoperatively, the timeslot was 14 days. For the 1-year and 2-year PROMs, the submission deadline was extended to 30 days. Notification emails (reminders) to complete the preoperative PROMs were automatically sent whenever a questionnaire was not completed. The notification system was standardized across the entire hospital, so this could not be modified for the purposes of this pilot. Patients received the first reminder at 6:00 PM on the day following the scheduling of their initial outpatient clinic appointment. The second reminder was sent five days before a scheduled hospital appointment (including in-person or telephone follow-ups), or if an information leaflet became available on the patient portal. No reminders were triggered after the postoperative PROMs had been sent. Since the preoperative consultation and first postoperative telephone follow-up were conducted by one of the physicians of the surgical department, these appointments provided opportunities to discuss the PROMs outcomes, make adjustments if necessary, or assist patients in completing the surveys if they had not done so already. Patients’ responses were not actively monitored, meaning that the treating physician did not act upon the submitted answers. Patients were advised to schedule an appointment if they experienced any abnormal postoperative complaints.

### Outcomes

The primary outcome was response rate of PROMs questionnaires, which was evaluated based on the proportion of self-reported responses to the PROMs at each follow-up time point. Based on recommendations of the PROMs Working Group of the International Society of Arthroplasty Registries (ISAR), a threshold of 60% was considered a sufficient response rate [15]. Secondary outcomes included preoperative and short-term postoperative physician-assisted response rates, combined patient- and physician-assisted response rates, responders to all five PROMs questionnaires, and between-group differences of responders versus non-responders at 6–8 weeks postoperatively.

### Statistical analysis

Statistical analyses were conducted using R Statistical Software (version 4.2.2) and RStudio (version 2026.01.1; R Core Team). The primary outcome is presented using frequencies and percentages. Secondary outcomes involving binomial and categorical data are reported with the same descriptives. Categorical variables of responders versus non-responders are compared with the chi-squared test. Non-normally distributed answers were compared between these groups using a Wilcoxon rank-sum test. A student-t test was used for normally distributed data. Paired groups (self-reported vs. physician-assisted) were analysed with the McNemar test.

## Results

**Fig. 1:**
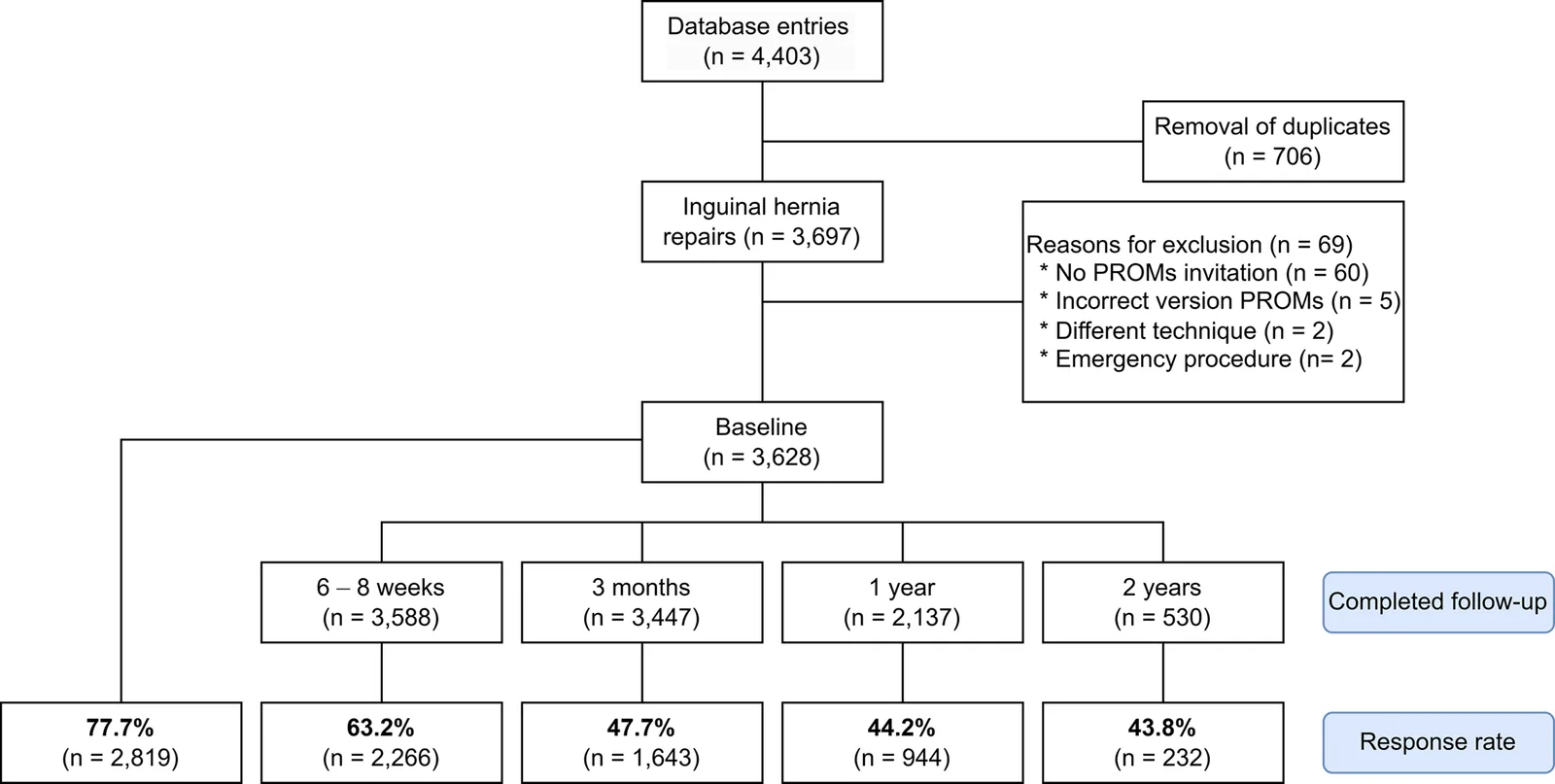
Flowchart of inclusions

Between January 18, 2023, and August 25, 2025, a total of 4,403 entries were recorded in the database. After removing duplicates, 3,697 patients who underwent inguinal hernia surgery and were invited to complete the PROMs questionnaires, remained. Following the screening, 3,628 individual patients were included in the final analysis. During the initial phase of PROMs implementation, the questionnaire version was updated and invitations were sent manually. As a result, several patients did not receive a questionnaire or received an incorrect version. All reasons for exclusion are illustrated in Fig. 1.

The majority of included patients were male (93.8%) with a median age of 62 years (IQR: 50–71). Regarding the surgical procedure, the TEP versus Lichtenstein ratio was 94:6. In terms of preoperative health status, 87.3% were ASA I or II. All baseline characteristics are summarized in Table 2.

**Table 2:** Baseline characteristics

No. of patients	3,628
Sex, n (%)	
Male	3,402 (93.8)
Female	226 (6.2)
Age, median (IQR), years	62.0 (50.0–71.0)
BMI, mean (SD), kg/m^2^	24.9 (3.1)
Procedure, n (%)	
TEP repair, unilateral	2,771 (76.4)
TEP repair, bilateral	627 (17.3)
Lichtenstein repair, unilateral	214 (5.9)
Lichtenstein repair, bilateral	12 (0.3)
Combined^a^	4 (0.1)
ASA, n (%)	
I	1,361 (37.5)
II	1,806 (49.8)
III	441 (12.2)
IV	20 (0.5)
Operative time, median (IQR), minutes	14.0 (11.0–20.0)

*IQR* interquartile range, *BMI* body mass index, *SD* standard deviation, *TEP* totally extraperitoneal, *ASA* American Society of Anaesthesiologist

^a^TEP repair combined with contralateral Lichtenstein procedure

### Response rates

Response rates for the preoperative and short-term postoperative PROMs questionnaires were 77.7% and 63.2%, respectively. Over time, response rates declined to 47.7%, 44.2%, and 43.8% at subsequent time points (Fig. 1).

Physician-assisted response rates were significantly higher than the self-reported rates (*p* < 0.001) for both the preoperative (87.9% vs. 77.7%) and short-term postoperative PROMs (79.2% vs. 63.2%). When physician-assisted and self-reported responses were combined, overall response rates increased further, reaching 97.3% preoperatively and 86.8% at short-term follow-up.

**Fig. 2:**
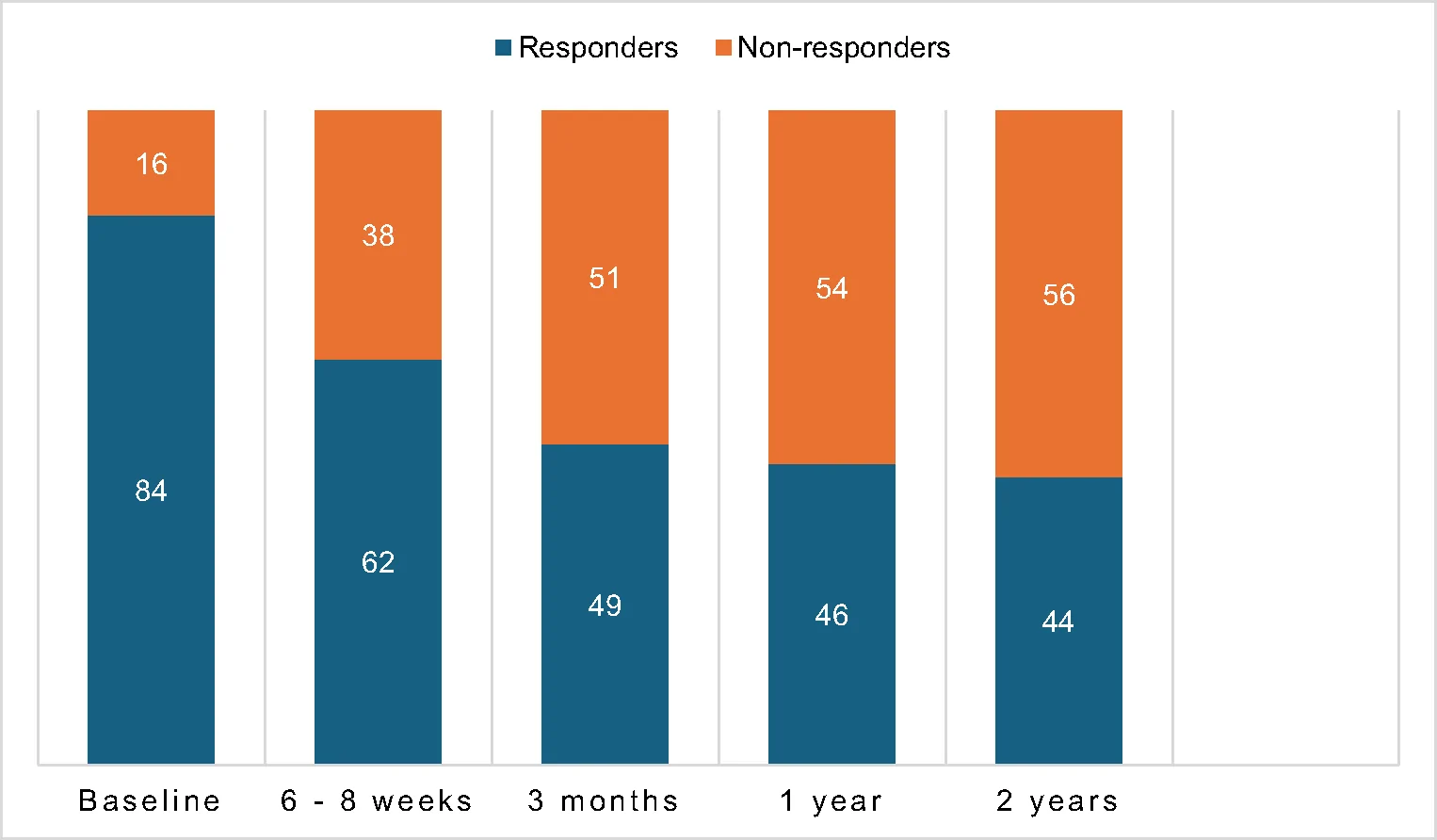
Response rates per time point for patients with ≥ 2 years of follow-up (*n* = 530)

Of the 530 patients who reached the 2-year postoperative follow-up, 22.8% answered all five questionnaires. Their response rates are shown in Fig. 2.

The answers to the preoperative and 6–8 weeks postoperative PROMs questionnaires are summarized in the Online Resource 1 (eTables 2 and 3).

## Responders vs. non- responders

Baseline characteristics were compared between responders and non-responders at short-term follow-up. Factors associated with non-response included younger age and a higher burden of comorbidities (ASA-score), as shown in Table 3.

**Table 3:** Responders vs. non-responders at 6–8 weeks postoperatively

	Responders (*n* = 2,266)	Non-responders (*n* = 1,322)	*p*-value
Sex, *n* (%)			0.684
Male	2,128 (93.9)	1,237 (93.6)	
Female	138 (6.1)	85 (6.4)	
Age, median (IQR), years	63.0 (52.0–71.0)	60.0 (47.0–72.0)	**< 0.001**
BMI, mean (SD), kg/m^2^	24.9 (3.1)	25.0 (3.2)	0.449
ASA, *n* (%)			**0.012**
I	843 (37.2)	503 (38.0)	
II	1,160 (51.2)	630 (47.7)	
III	256 (11.3)	177 (13.4)	
IV	7 (0.3)	12 (0.9)	

*IQR*, interquartile range; *BMI*, body mass index; *SD*, standard deviation; *ASA*, American Society of Anaesthesiologists

## Discussion

This study demonstrated feasibility of the preoperative and short-term postoperative (6–8 weeks) automated PROMs registration, as response rates were above 60% (77.7% and 63.2%, respectively). However, an overall declining response rate was observed at longer follow-up time points, with a decrease to 47.7% at 3 months, 44.2% at 1 year, and 43.8% at 2 years postoperatively, indicating a progressive reduction in patient engagement with longer follow-up. The highest rates were observed when the patient- and physician-assisted responses rates were combined. Baseline patient factors associated with short-term non-response included younger age and a higher burden of comorbidities.

Population-level outcomes following inguinal hernia surgery in the Netherlands remain unclear, as existing data primarily come from selected centres that perform research. Furthermore, these studies focus mainly on ‘traditional’ surgical outcomes, such as reoperations, hospital stays, etc [25, 26]. Currently, there has been a shift towards more patient-centred care and redefining surgical success for benign procedures. Rosenberg et al. propose to complement the traditional outcome measures with PROMs to reflect what matters most to patients, such as pain, quality of life, physical functioning, and satisfaction [16]. So far, other hernia registries have not yet routinely incorporated PROMs as part of their registry. In a limited approach, the Swedish Hernia Register (SHR) used four questions to assess PROMs regarding pain and patient satisfaction one year after surgery between 2012 and 2018. The first question evaluated CPIP and was derived from the validated Inguinal Pain Questionnaire (IPQ). It required patients to grade the maximum pain intensity experienced in the operated groin during the preceding week [17–19]. Non-responders were reminded within 30 days and answers were either returned by post or entered in an internet-based program. If patients had not responded to the questionnaire or the reminder, a telephone interview was planned. Notably, response rates of 70% and 75% were observed [18]. Inherently, paper-based systems increase administrative workload, a challenge also seen in the SHR, where clinical data are manually recorded by their surgeons. In contrast, our study benefits from automated PROMs collection, reducing this burden and increasing efficiency. Another advantage is that automated online questionnaires are more environmentally sustainable than traditional paper-based versions. While the information gained from this single IPQ question is useful, it is somewhat limited in scope [17, 18]. A very recent publication from the SHR mentioned the development of a new instrument to combine both pre- and postoperative patient-related outcomes [6]. However, the details of this instrument remain unclear.

In the same context, the Danish Hernia Database recently published a study protocol outlining its plan to integrate PROMs and link them with clinical data from the registry [8]. As part of a nationwide survey, Danish patients who underwent inguinal hernia repair will receive a Groin Hernia-Q (GHQ), an adaptation of the Abdominal Hernia-Q (AHQ) [20]. However, the English version of the GHQ has not yet been published, which limits direct comparisons with the PROMs used in our study [21]. The Danish GHQ demonstrated strong face validity, but psychometric validation has yet to be performed [22]. Its length (30 questions) may affect patient engagement, particularly during multiple follow-up time points, an issue not fully elaborated on in their study protocol. Their latest study showed that the GHQ scored around 25 on a scale of 100 for the QQ-10 survey question ‘the questionnaire was too long’ [22]. While lower scores are more favorable for this question, 25 is still considered quite high. To increase response rates, questionnaires should be kept short [23]. Nevertheless, by linking PROMs data with clinical data, the project aims to assess the long-term efficacy of different surgical approaches, a goal that is also pursued by the DIHA.

The decline in response rates over time is a well-documented phenomenon. Orthopaedic registries typically start with a mean response rate of 72% (SD: 21.8), which decreases to 57% (SD: 18.5) one year after surgery [24]. These rates can be increased by combining an automated system with manual collection, improving preoperative rates from 86% to 100% and postoperative responses at 1 year follow-up from 53% to 83% [25]. However, manual collection added €6 per surgical procedure to automated PROMs collection alone. It is questionable if the use of paper surveys to improve response rates is justified, as the response rate required for an adequate evaluation of treatment outcomes remains uncertain [24, 25].

Although multiple hernia registries have been successful [26–28], maintaining a registry comes with challenges, particularly the administrative burden and the difficulty of ensuring long-term follow-up. Variations in patient engagement can lead to incomplete data, potentially compromising the representativeness of the responding population, and thereby the validity of registry findings. The phenomenon of decreased patient engagement over time is illustrated by our study with the relatively low response rate of 44% after 2 years of postoperative follow-up. Patients experiencing pain are assumed to be more likely to respond to PROMs questionnaires, while those with no or minimal complaints may have less incentive to answer. Supporting this theory, a previous SHR study confirmed that responders reported higher rates of CPIP one year postoperatively than non-responders [18]. These selection and attrition biases raise concerns about representativeness of the responding population. Mommers et al. used the Core Outcome Measurements Index (COMI) to prospectively evaluate multiple patient-related outcomes including quality of life. They observed a higher-than-expected chronic pain rate of 14% following TEP repair. However, reported pain did not impact their perceived quality of life, which might be explained by the difficult interpretation of the questionnaire for both patient and physician [29]. Patient-specific factors can influence the response rate to questionnaires [30, 31]. Our study found that younger age and higher comorbidity burden may reduce response rates. Surprisingly, non-responders of the short-term PROMs were significantly younger, in contrast to our assumption that the online capturing of PROMs might be more complex for older and computer illiterate patients. We hypothesize that younger patients have reduced motivation to respond because they are (too) busy with numerous activities in their personal and professional life. A study conducted within the SHR also revealed that non-responders were an average of 7 years younger [32]. Furthermore, patients might perceive PROMs questionnaires as burdensome if their recovery was uncomplicated, which is more likely in younger patients. On the other hand Levens et al. demonstrated that older age may contribute to poor PROMs response rates. Therefore, patients with systemic comorbidities may not prioritize questionnaires on benign surgical outcomes [33]. Because age is associated with burden of disease, their association with non-response should be interpreted cautiously.

Wilcox et al. reviewed PROMs in RCTs to assess reporting quality and highlighted the lack of standardization [34]. They found that pain was examined in 99% of studies, with the Visual Analogue Scale (VAS) widely used to assess pain scores. However, many studies failed to specify the VAS instrument used or provide evidence of its validity [34]. This underscores the need for standardized PROMs reporting, as these data are crucial for guiding hernia management through comparison. As integrating automated PROMs seems feasible, the next step would be validation, ensuring data reliability and generalizability.

The current version of the PROMs has been reviewed by the Clinical Audit Board (CAB), whom were chosen by the Dutch Hernia Society (DHS) and Dutch association of surgeons (NVvH) [7]. The CAB consists of dedicated inguinal hernia surgeons. While the PROMs are concise and focused on just a few key domains, our current research shows that the phrasing could be improved in certain areas. For example, in the case of the preoperative PROMs, the questions about work and exercise were not posed to patients who were not experiencing pain, which resulted in missing baseline parameters. A suggestion would be to add the following options to the answers of Q1.3 and Q1.5 (Online Resource 1, eTable 1): “I am not employed” and “I never exercise”. By doing so, the timing of the answers (pre- or postoperative) has less influence, as these answers may change over time. For example, at baseline a patient might answer he or she does not work, while at 6–8 weeks of postoperative follow-up the patient has found a job. Additionally, there is variability in the answers to the pain-related question as data showed that some patients answered positively but still reported a NRS of 0, implying they have no pain. Conversely, patients who answered negatively were not given the opportunity to provide an NRS score, even though they might have scored their discomfort greater than NRS 0. Once a sufficient number of patients have reached the 2-year PROMs follow-up, the CAB will review and revise the questions to ensure that only relevant data are retained.

A major strength of this pilot is that patients completed PROMs independently, without any healthcare personnel interference, ensuring the self-reported responses were solely based on their own initiative. Although the automated PROMs system was considered successful overall, areas for improvement became clear when the study had already commenced. The number of duplicate records was substantial because each entry made on a different date in the electronic medical record resulted in duplicated data within the PROMs database. This also occurred when patients received multiple preoperative questionnaires, for example when a patient was initially treated with a watchful waiting strategy for an asymptomatic hernia but later experienced symptoms and scheduled a new appointment to discuss surgery. Another technical problem was the inability of automatically sending PROMs to patients who were operated for a recurrent hernia during the 2-year follow-up period after the initial procedure. An additional consequence of this limitation was that each patient was included only once in the final analyses. These technical issues, combined with the exclusion of patients who received no PROMs invitation or an incorrect version, may lead to a small overestimation of the true feasibility of the automated PROMs collection system. Other limitations include potential selection and attrition biases and varying patient interpretations of the PROMs questions. Future studies should focus on reasons for non-responding and improving patient engagement, such as contacting patients beforehand and integrating postoperative reminders [23].

## Conclusions

As far as we know, this pilot study is the first to describe the use of automated questionnaires for PROMs registration as part of routine clinical care in inguinal hernia surgery. The results are promising, with sufficient response rates at baseline and short-term follow-up, but highlight the challenge of declining response rates over time. Long-term automated PROMs collection has not yet been shown to achieve acceptable response rates. This is particularly problematic, as long-term outcomes are often the most relevant data for registry-based quality assessment. Therefore, strategies to optimize response rates and representativeness of the responding population should be explored. Integrating this approach into a national inguinal hernia registry has the potential to improve data collection and overall efficiency compared to current unautomated methods.

## Supplementary Information

Below is the link to the electronic supplementary material.

Supplementary Material 1

Supplementary Material 2
