# Supplementary material for: Feasibility of automated registration of Patient-Reported Outcome Measures (PROMs) for inguinal hernia surgery : a single-centre pilot study
Source: Hernia. 2026 Oct 15;30(1):374. doi: 10.1007/s10029-026-03822-4 (PMC13672378; doi:10.1007/s10029-026-03822-4)
Supplement: Supplementary file 1 [file 10029_2026_3822_MOESM1_ESM.pdf]

Online Resource 1

eTable 1 English translation of patient-reported outcome measures (PROMs) questionnaires

**Questions**	**Answers**	**Dynamics**
**Preoperative**		
1.1. Are you experiencing pain related to your inguinal hernia?	Yes / no	Fixed
1.2. What was the worst pain you experienced during the past week on a scale of 0 – 10? (0 = no pain, 10 = worst possible pain)	NRS 0 – 10	Fixed
1.3. Do you have paid work?	Yes / no	If Q1.1 yes
1.4. Which statement about work applies to you?	▪ I am fully able to work with my inguinal hernia.<br>▪ I work reduced or adjusted hours due to my inguinal hernia.<br>▪ I no longer work due to my inguinal hernia.	If Q1.3 yes
1.5. Do you exercise?	Yes / no	If Q1.1 yes
1.6. Which statement about exercising applies to you?	▪ I am fully able to exercise with my inguinal hernia.<br>▪ I exercise less or have adjusted my exercise due to my inguinal hernia.<br>▪ I no longer exercise due to my inguinal hernia.	If Q1.5 yes
1.7. Have you had previous surgery in your lower abdomen?	Yes / no	Fixed
1.8. Have you had surgery for an inguinal hernia on the same side before?	Yes / no	Fixed
1.9. In which hospital were you treated for this inguinal hernia?	Dropdown list of Dutch hospitals	If Q1.8 yes
**Postoperative 6 – 8 weeks**		
2.1. Are you experiencing pain related to your inguinal hernia repair?	Yes / no	Fixed
2.2. What was the worst pain you experienced during the past week on a scale of 0 – 10? (0 = no pain, 10 = worst possible pain)	NRS 0 – 10	Fixed
2.3. Are you able to work after your inguinal hernia repair?	Yes / no	If Q1.3 yes
2.4. How many days after your surgery could you return to work?	Open answer: number of days	If Q2.3 yes
2.5. Are you able to exercise after your inguinal hernia repair?	Yes / no	If Q1.5 yes
2.6. How many days after your surgery could you return to exercising?	Open answer: number of days	If Q2.5 yes
2.7. Have you undergone a re-operation on the same groin within the last 6 weeks?	Yes / no	Fixed
2.8. At which hospital were you re-operated?	Dropdown list of Dutch hospitals	If Q2.7 yes
**Postoperative 3 months**		
3.1. Are you experiencing pain related to your inguinal hernia repair?	Yes / no	Fixed
3.2. What was the worst pain you experienced during the past week on a scale of 0 – 10? (0 = no pain, 10 = worst possible pain)	NRS 0 – 10	If Q3.1 yes

3.3. Have you undergone a re-operation on the same groin within the last 3 months?	Yes / no	Fixed
3.4. At which hospital were you re-operated?	Dropdown list of Dutch hospitals	If Q3.3 yes
3.5. Are you satisfied with your treatment?	Yes / no	Fixed
3.6. What score would you give your treatment? (0 = very dissatisfied, 10 = very satisfied)	NRS 0 – 10	Fixed
3.7. If you had to choose again, would you still opt for surgery for your inguinal hernia?	Yes / no	Fixed
**Postoperative 1 year**		
4.1. Are you experiencing pain related to your inguinal hernia repair?	Yes / no	Fixed
4.2. What was the worst pain you experienced during the past week on a scale of 0 – 10? (0 = no pain, 10 = worst possible pain)	NRS 0 – 10	If Q4.1 yes
4.3. Have you undergone a re-operation on the same groin within the last year?	Yes / no	Fixed
4.4. At which hospital were you re-operated?	Dropdown list of Dutch hospitals	If Q4.3 yes
**Postoperative 2 years**		
5.1. Are you experiencing pain related to your inguinal hernia repair?	Yes / no	Fixed
5.2. What was the worst pain you experienced during the past week on a scale of 0–10? (0 = no pain, 10 = worst possible pain)	NRS 0 – 10	If Q5.1 yes
5.3. Have you undergone a re-operation on the same groin within the last 2 years?	Yes / no	Fixed
5.4. At which hospital were you re-operated?	Dropdown list of Dutch hospitals	If Q5.3 yes

NRS, numeric rating scale; Q, question

eTable 2a Preoperative fixed PROMs (n = 2,819), presented as n (%)

Pain	2,062 (73.1)
Pain NRS categories[a]	
No pain	766 (27.2)
Mild pain	360 (12.8)
Moderate pain	1,243 (44.0)
Severe pain	450 (16.0)
Previous abdominal surgery	979 (34.7)
Recurrence	240 (8.5)

PROMs, patient-reported outcome measures; NRS, numeric rating scale
[a] No pain NRS 0; mild pain NRS 1 – 3; moderate pain NRS 4 – 7; severe pain NRS 8 – 10

eTable 2b Preoperative dynamic PROMs domain: physical functioning (n = 2,062), presented as n (%)

Working	1,276 (61.9)
Work statement	
I am fully able to work with my inguinal hernia	927 (45.0)
I work reduced or adjusted hours due to my inguinal hernia	305 (14.8)
I no longer work due to my inguinal hernia	44 (2.1)
Not working	786 (38.1)
Exercising	1,138 (55.2)
Exercise statement	
I am fully able to exercise with my inguinal hernia	268 (13.0)
I exercise less or have adjusted my exercise due to my inguinal hernia	627 (30.4)
I no longer exercise due to my inguinal hernia	243 (11.8)
Not exercising	924 (44.8)

PROMs, patient-reported outcome measures

eTable 3a Postoperative fixed PROMs at 6 – 8 weeks postoperatively (n = 2,266), presented as n (%)

Pain	754 (33.3)
Pain NRS categories[a]	
No pain	1,534 (67.7)
Mild pain	311 (13.7)
Moderate pain	358 (15.8)
Severe pain	63 (2.8)
Recurrence	8 (0.4)

PROMs, patient-reported outcome measures; NRS, numeric rating scale;
[a] No pain NRS 0; mild pain NRS 1 – 3; moderate pain NRS 4 – 7; severe pain NRS 8 – 10

eTable 3b Postoperative dynamic PROMs at 6 – 8 weeks postoperatively

Resumed working[a], n (%)	1,351 (96.8)
Return to work[a], median (IQR), days	7.0 (5.0 – 14.0)
Resumed exercising[b], n (%)	1,188 (89.9)
Return to exercise[b], median (IQR), days	14.0 (14.0 – 21.25)

IQR, interquartile range
[a] n = 1,396
[b] n = 1,321
