# Supplementary material for: Feasibility of automated registration of Patient-Reported Outcome Measures (PROMs) for inguinal hernia surgery : a single-centre pilot study
Source: Hernia. 2026 Oct 15;30(1):374. doi: 10.1007/s10029-026-03822-4 (PMC13672378; doi:10.1007/s10029-026-03822-4)
Supplement: Supplementary file 2 [file 10029_2026_3822_MOESM2_ESM.pdf]

Online Resource 2

eTable 1 Nederlandse versie PROMs vragenlijsten

**Vragen**	**Antwoorden**	**Variabelen**
**Preoperatief**		
1.1. Heeft u pijn van uw liesbreuk?	Ja / nee	Standaard
1.2. Wat was de ergste pijn in de afgelopen week op een schaal van 0 – 10? (0 = geen pijn, 10 = ergst mogelijke pijn)	NRS 0 – 10	Standaard
1.3. Werkt u?	Ja / nee	Indien ja op 1.1
1.4. Welke stelling is voor u van toepassing? (werk)	▪ Ik werk volledig met mijn liesbreuk.<br>▪ Ik werk minder/ aangepast door mijn liesbreuk.<br>▪ Ik werk niet meer door mijn liesbreuk.	Indien ja op 1.3
1.5. Sport u?	Ja / nee	Indien ja op 1.1
1.6. Welke stelling is voor u van toepassing? (sport)	▪ Ik sport volledig met mijn liesbreuk.<br>▪ Ik sport minder/ aangepast door mijn liesbreuk.<br>▪ Ik sport niet meer door mijn liesbreuk.	Indien ja op 1.5
1.7. Bent u eerder in de onderbuik geopereerd?	Ja / nee	Standaard
1.8. Bent u eerder aan een liesbreuk aan dezelfde lies geopereerd?	Ja / nee	Standaard
1.9. In welk ziekenhuis bent u geopereerd?	Keuzelijst Nederlandse ziekenhuizen	Indien ja op 1.8
**Postoperatief 6 – 8 weken**		
2.1. Heeft u pijn na uw liesbreukoperatie?	Ja / nee	Standaard
2.2. Wat was de ergste pijn in de afgelopen week op een schaal van 0 – 10? (0 = geen pijn, 10 = ergst mogelijke pijn)	NRS 0 – 10	Standaard
2.3. Kunt u werken na uw liesbreukoperatie?	Ja / nee	Indien ja op 1.3
2.4. Hoeveel dagen na uw liesbreukoperatie kon u weer werken?	Open antwoord: aantal dagen	Indien ja op 2.3
2.5. Kunt u sporten na uw liesbreukoperatie?	Ja / nee	Indien ja op 1.5
2.6. Hoeveel dagen na uw liesbreukoperatie kon u weer sporten?	Open antwoord: aantal dagen	Indien ja op 2.5
2.7. Bent u de afgelopen 6 weken opnieuw geopereerd aan dezelfde lies?	Ja / nee	Standaard
2.8. In welk ziekenhuis bent u geopereerd?	Keuzelijst Nederlandse ziekenhuizen	Indien ja op 2.7
**Postoperatief 3 maanden**		
3.1. Heeft u pijn na uw liesbreukoperatie?	Ja / nee	Standaard
3.2. Wat was de ergste pijn in de afgelopen week op een schaal van 0 – 10? (0 = geen pijn, 10 = ergst mogelijke pijn)	NRS 0 – 10	Indien ja op 3.1
3.3. Bent u in de afgelopen 3 maanden opnieuw geopereerd aan dezelfde lies?	Ja / nee	Standaard

3.4. In welk ziekenhuis bent u geopereerd?	Keuzelijst Nederlandse ziekenhuizen	Indien ja op 3.3
3.5. Bent u tevreden over uw behandeling?	Ja / nee	Standaard
3.6. Wat voor cijfer geeft u voor uw behandeling op een schaal van 0 – 10? (0 = zeer ontevreden, 10 = zeer tevreden)	NRS 0 – 10	Standaard
3.7. Als u opnieuw mocht kiezen om geopereerd te worden aan de liesbreuk, zou u zich dan alsnog laten opereren?	Ja / nee	Standaard
**Postoperatief 1 jaar**		
4.1. Heeft u pijn na uw liesbreukoperatie?	Ja / nee	Standaard
4.2. Wat was de ergste pijn in de afgelopen week op een schaal van 0 – 10? (0 = geen pijn, 10 = ergst mogelijke pijn)	NRS 0 – 10	Indien ja op 4.1
4.3. Bent u in het afgelopen jaar opnieuw geopereerd aan dezelfde lies?	Ja / nee	Standaard
4.4. In welk ziekenhuis bent u geopereerd?	Keuzelijst Nederlandse ziekenhuizen	Indien ja op 4.3
**Postoperatief 2 jaar**		
5.1. Heeft u pijn na uw liesbreukoperatie?	Ja / nee	Standaard
5.2. Wat was de ergste pijn in de afgelopen week op een schaal van 0 – 10? (0 = geen pijn, 10 = ergst mogelijke pijn)	NRS 0 – 10	Indien ja op 5.1
5.3. Bent u in de afgelopen 2 jaar opnieuw geopereerd aan dezelfde lies?	Ja / nee	Standaard
5.4. In welk ziekenhuis bent u geopereerd?	Keuzelijst Nederlandse ziekenhuizen	Indien ja op 5.3

PROMs, patient-reported outcome measures; NRS, numeric rating scale
